# Phylogeny of the nematode genus *Pristionchus *and implications for biodiversity, biogeography and the evolution of hermaphroditism

**DOI:** 10.1186/1471-2148-7-104

**Published:** 2007-07-02

**Authors:** Werner E Mayer, Matthias Herrmann, Ralf J Sommer

**Affiliations:** 1 Max Planck Institute for Developmental Biology, Department of Evolutionary Biology, Spemannstr. 37, 72076 Tübingen, Germany

## Abstract

**Background:**

The nematode *Pristionchus pacificus *has originally been developed as a satellite organism for comparison to *Caenorhabditis elegans*. A 10X coverage of the whole genome of *P. pacificus *is available, making *P. pacificus *the first non-*Caenorhabditis *nematode with a fully sequenced genome. The macroevolutionary comparison between *P. pacificus *and *C. elegans *has been complemented by microevolutionary studies of closely related strains and species within the genus *Pristionchus*. In addition, new understanding of the biology of *Pristionchus *from field studies, demonstrating a close association with various scarab beetles and the Colorado potato beetle, supports consideration of this nematode in studies of ecosystems. In the course of field studies on four continents more than 1,200 isolates were established from 15,000 beetle specimens representing 18 *Pristionchus *species. Two remarkable features of the *Pristionchus *– beetle association are the high species specificity of the interaction and the interception of the beetle's sex communication system for host recognition by the nematodes, as suggested by chemotaxis studies. Evolutionary interpretations of differences in developmental, behavioral and ecological patterns require a phylogenetic framework of the genus *Pristionchus*.

**Results:**

Here, we provide a robust phylogeny of all 18 available *Pristionchus *species based on a set of 27 ribosomal protein genes encompassing a total of 10,971 bp. The phylogenetic tree provides evidence for North American and European clades, which are embedded in a deeper clade that includes Asian species. It also indicates putative invasion events. Of the 18 *Pristionchus *species, 13 are gonochoristic and five are hermaphroditic. The phylogeny indicates that all hermaphroditic species have arisen independently within the genus *Pristionchus*.

**Conclusion:**

Combined ribosomal protein cDNA data can provide the basis for reconstruction of a robust phylogenetic framework for microevolutionary and biogeographic analyses. An additional major implication of our studies is the use of *Pristionchus *for nematode biodiversity assessments. While some species are represented by more than 100 isolates, others were found less than four times. Such patterns were observed on all continents and in all phylogenetic clades indicating that species asymmetry is a widespread phenomenon, which can now be further investigated by molecular tools.

## Background

The nematode *Pristionchus pacificus *is a laboratory model organism to study developmental biology, evolutionary biology, and behavior in comparative context to other nematodes such as *Caenorhabditis elegans *(for review see [1]). Recent studies indicate a close association of these nematodes to various scarab beetles and the Colorado potato beetle (CPB), thus extending the knowledge of *Pristionchus *ecology [2-4]. For example, *P. pacificus *is found on the Oriental beetle *Exomala orientalis *in Japan and the United States, whereas two common European species *P. maupasi *and *P. entomophagus *are predominantly found on cockchafers (*Melolontha *ssp.) and dung beetle (*Geotrupes *ssp.), respectively [2,4]. Surprisingly, these studies revealed an notable precision in the species-specificity between *Pristionchus *nematodes and beetles. Chemotaxis studies indicated that *Pristionchus *olfaction differs strongly between closely related species and may be involved in shaping the nematode-host interactions [1,5].

The genus *Pristionchus *belongs to the family Diplogastridae, a monophyletic group within the Rhabditidae that encompasses some 300 described free-living species in 28 genera [6,7]. Most diplogastrids are gonochoristic; hermaphrodites are described only in two genera, *Diplogasteroides *and *Pristionchus*. *P. pacificus *and *C. elegans *belong both to clade V nematodes [8,9]. Clade V encompasses *C. elegans *and other members of the suborder Rhabditina with the vertebrate-parasitic order Strongylida, the entomopathogenic genus *Heterorhabditis *and the order Diplogasterida. The latter, which includes *Pristionchus*, is a sister group of some rhabditids [8]. They resemble each other in overall morphology, culture conditions, mode of reproduction and other features, i.e. they both feed on *Escherichia coli *bacteria under laboratory conditions and have a generation time of 3–4 days at 20°C. They develop by lineage-dependant differentiation through a fixed number of cells and pass through four juvenile stages. Both species are self-fertilizing hermaphrodites with a XX genotype. However, the rare occurrence of spontaneous males of the XO genotype generated by X-chromosomal non-disjunction provides a way for outcrossing [10,11].

Despite the many similarities, a number of differences and peculiarities exist between *P. pacificus *and *C. elegans *that are subject to current investigations. Diplogastrids differ from rhabditids in a number of morphological, developmental and behavioral aspects, e.g., by the absence of a grinder in the terminal bulb, the J1 to J2 moult that occurs in diplogastrids within the egg before hatching, or chemoattraction profiles [5-7]. Description of vulva and gonad development at the cellular level revealed substantial differences in the cell-cell interactions and the genetic and molecular networks regulating developmental processes [12-14].

To complement the developmental and genetic studies in *P. pacificus*, a genetic linkage map and a physical map have been generated and a whole genome sequencing draft of *P. pacificus *is available [15-18]. In addition, the genomes of two sister species, *P. maupasi *and *P. entomophagus*, have been sequenced at low coverage.

Field studies in the years 2004 – 2006 in western Europe, North America, South Africa and Japan provided a first overview of the beetle-associated *Pristionchus *fauna in the respected areas [2-4,19]. In the course of these global surveys, 15 distinct *Pristionchus *species were discovered (Table 1). Six of them could be identified as the known species *P. pacificus*, *P. maupasi*, *P. uniformis*, *P. entomophagus*, *P. lheritieri*, and *P. aerivorus *and four others represented novel species, which were described as *P. pseudaerivorus*, *P. marianneae*, *P. pauli*, and *P. americanus *[2,3]. Five other species could not be matched to valid species names; two of them from western Europe (*P*. sp. 4, *P*. sp. 6) and three from Japan (*P*. sp. 11, *P*. sp. 14, *P*. sp. 15) [2,5].

**Table 1:** Distribution of *Pristionchus *species

**Species**	**Major origin of isolates**	**Other locations**	**Number of isolates**	**Reference strain**	**Original Publication**
P. *pacificus*	Japan	USA, South Africa, and worldwide	> 130	PS312	[49]
*P*. sp. 11	Japan		2	RS5228	[4]
P. *maupasi*	western Europe		> 70	RS0143	[50]
P. *aerivorus*	North America		83	RS5106	[51]
*P. pseudaerivorus*	North America		71	RS5139	[3]
P. *americanus*	North America		3	RS5140	[3]
P. *marianneae*	North America		45	RS5108	[3]
*P. pauli*	North America		35	RS5130	[3]
*P*. sp. 3	North America		1	CZ3975	this study
P. *lheritieri*	western Europe		24	SB245	[52]
*P. uniformis*	western Europe	North America	> 160	RS0141	[53]
*P*. sp. 13	Romania		4	RS5231	this study
*P. entomophagus*	western Europe	North America, New Zealand	> 240	RS0144	[54]
*P*. sp. 4	western Europe		2	RS5050	[2]
*P*. sp. 6	western Europe		11	RS5101	[2]
*P*. sp. 10	Nepal		2	RS5133	this study
*P*. sp. 14	Japan		1	RS5230	[4]
*P*. sp. 15	Japan		2	RS5229	[4]

**Figure 1:**
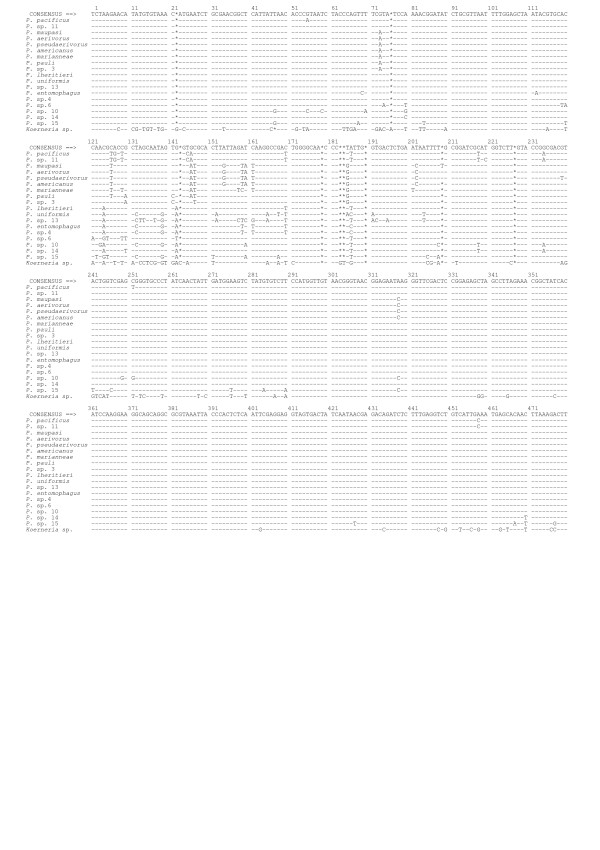
***Pristionchus SSU *sequence alignment**. Sequences of 18 *Pristionchus *species and *Koerneria *sp. were aligned manually. Dashes (-) indicate identity to the majority consensus sequence at the top; asterisks (*) indicate alignment gaps. Numbering refers to the *SSU *segment obtained in this study. The sequences are available from the GenBank database under the accession codes [GebBank:DQ270018] to [GebBank:DQ270025], [GebBank:DQ419900] to [GebBank:DQ419904], [GebBank:EF216858] to [GebBank:EF216860], and [GenBank:EF623995] to [GenBank:EF623997].

Species determination of large numbers of nematode isolates from extensive field studies requires a fast, reliable and easy procedure. This can be achieved by combining morphological identification of new specimen with easily obtainable molecular taxonomic markers. We choose to apply a 471 bp segment of the of the 5' part of the small subunit ribosomal RNA gene (*SSU*) for this purpose as described by Blaxter et al., Floyd et al. and Herrmann et al. [2-4,8,20]. Briefly, gravid female nematodes were isolated to establish isogenic female lines. Single offspring worms were picked, lysed and subjected to *SSU*-specific PCR amplification. The resulting fragments were sequenced directly and their sequences compared to those of *Pristionchus *species reference strains (Figure 1). A sequence matching one of a reference strain suggested identical species. The species identification was then verified by crossing the new isolates and reference strains to produce viable and fertile offspring. Three observations were made: First, all isolates of a given species had invariably identical *SSU *sequences. Second, single nucleotide differences (substitutions or indels) indicated distinct species (as verified by mating experiments) and not intra-specific variability (e.g. *P. aerivorus *and *P. americanus*) [3]. All observed differences appeared to be fixed differences between species. Third, in one case a cryptic species pair could not be distinguished by the *SSU *sequence but only by their mode of reproduction and by mating experiments (*P. maupasi *and *P. aerivorus*). Thus, the *SSU *proved to be a powerful tool for species identification within the genus *Pristionchus*.

The current research in *P. pacificus *developmental biology, behavior, ecology and microevolution requires a detailed knowledge of the phylogenetic relationships within the genus *Pristionchus*, the Diplogastridae family, as well as clade V nematodes as a whole. While Kiontke and Fitch have provided a detailed phylogeny of clade V nematodes in 2005, the phylogeny at the family and genus level has not been studied with molecular techniques [21]. The molecular analyses of the *SSU *sequences as provided by Herrmann et al. is not sufficient for this purpose since the data harbor only a limited number of informative characters and provide only a single gene-based phylogeny [2,3]. To obtain a robust phylogeny of all 18 *Pristionchus *species currently available in the laboratory we set out to obtain a molecular data set that should represent an easily accessible unbiased representation of the species' genomes. Here, we describe 27 ribosomal protein genes that were concatenated to yield a data set of 10,971 aligned nucleotides. Phylogenetic trees were constructed by various methods generating a robust phylogeny of the genus *Pristionchus*. Implications for biogeography, biodiversity and origin of hermaphroditism are discussed.

## Results

### Identification of three novel species

In soil samples collected during the field surveys in the years 2004–2006 and provided to us by colleagues, we identified three additional novel *Pristionchus *species (Table 1). These species were preliminarily designated as *P*. sp. 3, *P*. sp. 10, and *P*. sp. 13 and will be taxonomically described elsewhere. *Pristionchus *sp. 3 is a gonochoristic species, represented by the single strain CZ3975, which originated from a soil sample from a flowerbed at the Cold Spring Harbor Laboratory. It is characterized by the *SSU *sequence with the accession code [GenBank:EF623995]. Male and female worms of this species do not mate with any of the other 17 *Pristionchus *species in our collection. The hermaphroditic species *Pristionchus *sp. 10 was found in two isolates from soil samples from Tato Pani near Pokhara, Nepal. The reference strain is RS5133. Males and female worms mate successfully between the two strains of this species but produce no offspring with any of the other 17 *Pristionchus *species in our collection. *Pristionchus *sp. 10 is characterized by the *SSU *sequence with the accession code [GenBank:EF623996]. *Pristionchus *sp. 13 was isolated from a soil sample from Buftea near Bucharest, Romania. The reference strain is RS5231. It is characterized by the *SSU *sequence with the accession code [GenBank:EF623997]. Males and female worms of this species do not produce offspring with any of the other 17 *Pristionchus *species in our collection. With these three novel species, a total of 18 *Pristionchus *species, of which representative strains are kept as live stocks, has been isolated from beetle and soil samples on four different continents.

### Molecular markers for phylogenetic inference

An expanded multi-locus data set encompassing several independently evolving genes was obtained by sequence analysis of EST libraries and the identification of frequently expressed genes. Gene transcripts coding for ribosomal proteins proved to be easily accessible in cDNA preparations from all *Pristionchus *species and could be assumed to evolve with similar rates. Preliminary tests showed that intraspecific polymorphism was very low for these genes and did not obstruct phylogenetic signals (data not shown). A total of 27 ribosomal protein genes was selected and amplified by RT-PCR with gene-specific primers from all 18 *Pristionchus *species and from the *Koerneria *sp. strain RS1982 (see Additional file 1). *Koerneria *was used as an outgroup because of its close morphological relatedness to *Pristionchus*, thereby reducing long-branch attraction effects, and its availability and accessibility for experimental studies [6]. The PCR products were sequenced directly with PCR primers. The 5' cDNA ends of all but the *rpl-27 *gene in *Koerneria *sp. could be obtained with a primer specific for the trans-spliced leader SL1. The latter cDNA fragment was amplified using an SL2b primer. The resulting sequences were aligned manually. Since the first nucleotides at the 5' end could not be obtained in all cases the first codons were eliminated from further analyses as indicated in Table 2. Only coding sequences including the stop codons were used since the highly diverged 3' untranslated regions could not be aligned unambiguously.

**Table 2:** Ribosomal protein genes used in the phylogenetic analysis

**Ribosomal protein gene**	**Chromosomal assignment in *C. elegans***	**Number of 5' codons omitted from analysis**	**Coding nucleotides used for analysis**
*rpl-1*	III	3	642
*rpl-2*	V	0	783
*rpl-10*	II	5	630
*rpl-14*	I	5	402
*rpl-16*	III	5	597
*rpl-23*	III	5	408
*rpl-26*	II	3	423
*rpl-27*	I	6	393
*rpl-27a*	I	8	420
*rpl-28*	V	7	387
*rpl-29*	IV	0	189
*rpl-30*	I	4	339
*rpl-31*	I	8	345
*rpl-32*	II	8	381
*rpl-34*	IV	4	330
*rpl-35*	III	8	348
*rpl-38*	V	6	195
*rpl-39*	V	8	132
*rps-1*	III	6	762
*rps-8*	IV	3	618
*rps-14*	III	8	435
*rps-20*	I	0	357
*rps-21*	III	0	267
*rps-24*	IV	0	396
*rps-25*	IV	7	336
*rps-27*	V	0	255
*rps-28*	IV	0	201
			

Total number of nucleotides:			10,971

The 27 coding sequences consisted of 132 bp to 783 bp, encompassing a total of 10,971 aligned nucleotides. The number of variable sites between the *Pristionchus *sequences was 2725, out of which 1571 were parsimony-informative. Transitions were in excess over transversions at a ratio of Ts/Tv = 1.51 within the genus *Pristionchus*. The number of synonymous and non-synonymous substitutions was calculated by the modified Nei-Gojobori method with the help of the MEGA3.1 software resulting in average values of d_S _= 0.181 and d_N _= 0.021 [22-24]. Thus, there was a 8.6-fold excess of synonymous over non-synonymous substitutions indicative of purifying selection.

All putative coding sequences had open reading frames of conserved lengths except two genes with apparent premature stop codons. The *rpl-2 *sequence obtained from *P. entomophagus *had a nonsense mutation at nucleotide position 37 leading to a stop codon. Likewise, the *rps-8 *sequence of *P*. sp. 4 showed a stop codon at position 493 of the alignment. In both cases the remaining sequence was highly conserved indicating a recent inactivation of the genes. For subsequent phylogenetic analyses the complete alignable sequences were used.

Among the 10,971 bp available for each taxon the nucleotide assignment at some variable positions remained ambiguous. Most species showed one to eight such sites. The exceptions were *P. maupasi *with 28, *P. entomophagus *with 35, *P. marianneae *with 55, and *P*. sp. 14 with 64 ambiguous sites. These ambiguities could be verified in separate PCR and sequencing reactions, and they occurred predominantly at synonymous positions that were also variable between species. We concluded that these particular genes were in a heterozygous state and represent intraspecific polymorphisms or resulted from recent gene duplication in the reference strain of that species. In the subsequent analyses the sequences were included using the standard ambiguity codes and the sites were thus treated as being polymorphic.

Sequence comparisons between the taxa revealed uncorrected mean character differences of *Koerneria *to all *Pristionchus *species of 18.0 to 18.8%, whereas within the genus *Pristionchus *the concatenated ribosomal protein cDNA sequences differed by merely 1.7 – 10.3%. The most distant *Pristionchus *species was *P*. sp. 15. The majority of species differed by 7 to 8%, while the closest was the species pair *P. aerivorus *and *P. pseudaerivorus*, which differed by only 1.7%. Thus, most of the *Pristionchus *species showed comparable or smaller distances than the *Caenorhabditis *species *C. elegans *and *C. briggsae*, which differed by 8.6% when their orthologous ribosomal protein genes were included in the analysis.

### Molecular phylogeny of the genus *Pristionchus*

**Figure 2:**
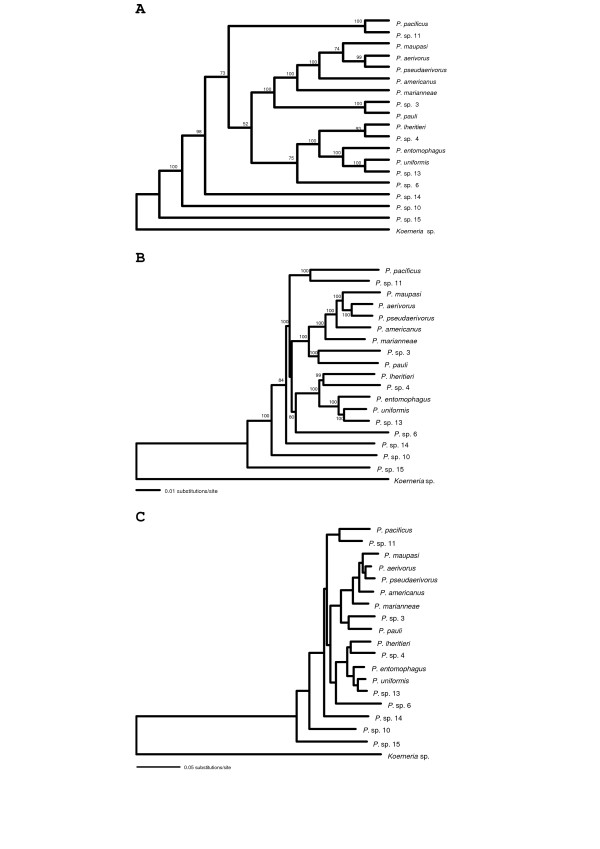
**Phylogenetic relationships of *Pristionchus* species based on ribosomal protein genes.** The phylogenetic trees of 18 *Pristionchus *species and *Koerneria *sp. were reconstructed from 10,971 bp of concatenated ribosomal protein CDS with the help of the PAUP*4.0b10 software [28]. **A**. Maximum parsimony tree obtained by the heuristic search algorithm. Multistate characters were interpreted as polymorphisms, gaps asfifth state. The tree was rooted at midpoint. Robustness of the tree topology was evaluated by 1000 bootstrap replications. The support values are shown at the nodes. **B**. Neighbor-joining tree. The tree was reconstructed using the BIONJ algorithm [29] and HKY distances [30]. The tree was rooted at midpoint. Numbers at nodes indicate bootstrap support values after 1000 replications. **C**. Maximum likelihood tree. The best substitution model, corresponding to the GTR+G+I model, was determined by Modeltest 3.7 [23] and the phylogenetic relationships were reconstructed by the heuristic search algorithm. The tree was rooted at midpoint.

The data set of multiple ribosomal protein genes was concatenated to produce a 10,971 bp-long super-gene alignment, which was then used for phylogenetic inference [25]. Various methods were applied. Prior to all analyses the best substitutions model for the *Pristionchus *data set (excluding *Koerneria *sp.) was selected by hierarchical likelihood ratio tests and the Akaike information criterion (AIC) in Modeltest 3.7 [26,27]. The selected model corresponded to GTR+G+I. Maximum parsimony analysis (MP) was performed in PAUP*4.0b10 using the heuristic search with stepwise-addition option [28]. Multistate taxa were interpreted as polymorphisms, gaps as fifth character state. Tree-bisection-reconnection (TBR) was chosen as branch swapping algorithm. Robustness of the tree topology was evaluated by 1000 bootstrap replications (Figure 2A). Neighbor-joining (NJ) analyses were performed in PAUP*4.0b10 using the BIONJ algorithm with the HKY85 distance option (Figure 2B) or with maximum likelihood distance settings as determined by Modeltest 3.7 [27,29,30]. In both cases the resulting tree topology was identical. The latter settings were also applied for ML analysis by heuristic search in PAUP*4.0b10 with TBR as branch swapping algorithm. The topology obtained by all three methods for phylogenetic reconstruction was identical (Figure 2C).

A significant grouping into three different clades is apparent. *Pristionchus pacificus *and *P*. sp. 11 fall into the same branch as sister species. The American species *P. aerivorus*, *P. pseudaerivorus*, *P. americanus*, *P. marianneae*, *P. pauli*, and *P*. sp. 3 group together with the European species *P. maupasi*. The other mostly European species *P. lheritieri*, *P. entomophagus*, *P. uniformis*, *P*. sp. 4, and *P*. sp. 13 fall into another distinct clade, which is joined by species *P*. sp. 6 as outlier. The species *P*. sp. 14, *P*. sp. 10 and *P*. sp. 15 from Asia are located in deeper clades in the phylogenetic tree.

While the bootstrap values provide very high support for the phylogenetic relationships within the three major clades, the nodes interconnecting the major clades appeared not to be significant. To obtain additional support for the topology a Bayesian analysis was performed with the help of the MrBayes 3.2.1 software for Macintosh [31]. The program was run for 150,000 generations, a total of 1,501 trees were sampled and after burning-in the trees 376–1501 were evaluated. The resulting phylogenetic consensus tree is displayed in Figure 4 and the posterior probabilities are shown at the nodes. The topology is identical to the MP, NJ, and ML trees and is highly supported by the posterior probability values. The only node that is not significantly resolved (posterior probability of 0.82) determines the position of the *P. pacificus *and *P*. sp. 11 clade. Here, in 18% of the sampled trees *P. pacificus *and *P*. sp. 11 are joined by *P*. sp. 14. Attempts to resolve this node by likelihood mapping with TREE-PUZZLE 5.2 by investigating various constellations of taxa did not improve our understanding of the branching pattern [32,33].

Although the major clades could be clearly identified by the molecular phylogenetic analysis, the nodes interconnecting these clades were less supported in MP and NJ phylograms and were separated by short branch lengths only. This seems to be due to stochastic segregation and incomplete lineage sorting of the ribosomal protein genes, which could result from ancestral speciation events in close succession from a polymorphic population and/or frequent hybridization and gene introgression before reproductive barriers were established.

## Discussion

Here, we present a first robust phylogeny of 18 available nematode species of the genus *Pristionchus *based on the analysis of 27 ribosomal protein genes totalling 10,971 bp. This molecular phylogeny provides a framework for mechanistic studies in developmental biology, evolution, behavior, and ecology.

### Evolution of hermaphroditism

The most common mode of reproduction of *Pristionchus *nematodes is by gonochorism, i.e. females with XX genotype and males with XO genotype have to mate to produce offspring. Thirteen of the species in our collection fall into this category. Five of the *Pristionchus *species, however, are self-fertilizing hermaphrodites, presumably of the XX genotype with males emerging at low frequency by X-chromosomal non-disjunction to enable occasional outcrossing. Among diplogastrid nematodes only one other genus harbors hermaphrodites, *Diplogasteroides*, which was also isolated on cockchafers together with *Pristionchus *species [7,34]. The diplogastrid species *Koerneria *sp., which according to Fürst von Lieven and Sudhaus (2003), was taken as outgroup in this study as a close relative to *Pristionchus*, is a gonochoristic species like *P*. sp. 15 from Japan, which appeared to diverge first in the *Pristionchus *genealogy [6].

**Figure 3:**
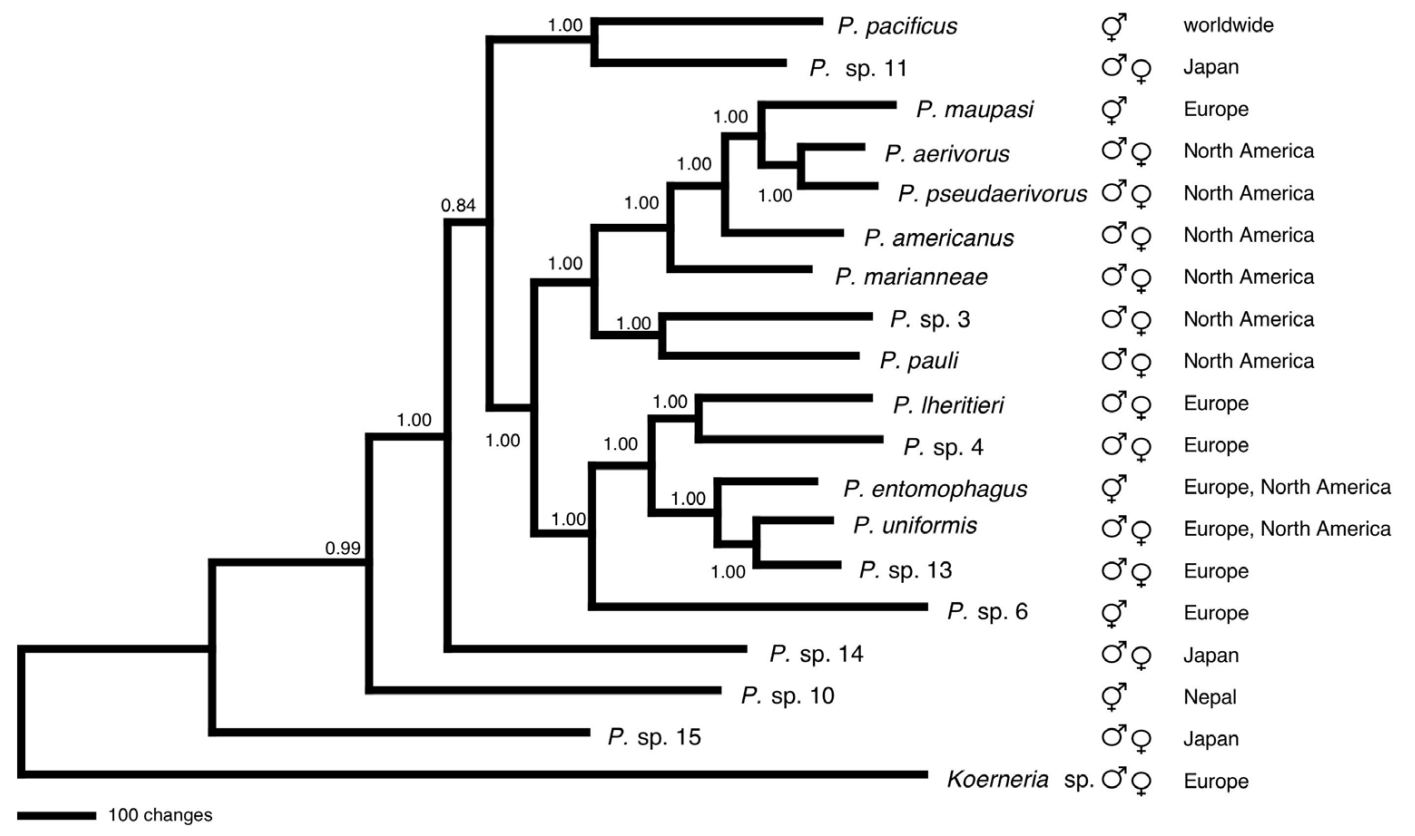
**Bayesian tree of *Pristionchus *species based on ribosomal protein genes**. The phylogenetic relationships were reconstructed from concatenated ribosomal protein CDS by Bayesian inference using the MrBayes 3.2.1 software [31]. The evolutionary model was set to GTR+I+G. The analysis was run for 150,000 generations, burn-in time was for 37,500 generations, sampling frequency was 1 in 100 generations. *Koerneria *sp. was included as the closest related genus to *Pristionchus*. The tree was rooted at midpoint. Numbers at nodes indicate posterior probabilities. Hermaphroditic and gonochoristic (male/female) species are marked. The geographic origins of the species are indicated.

The five hermaphroditic species all occur as terminal taxa in different clades of different geographic origin (Figure 3). This is a strong indication for multiple and independent origins of hermaphroditism in *Pristionchus *nematodes. These findings suggest a high plasticity in the sex determination system regulating hermaphroditic vs. female development. Furthermore, only a small number of mutations, which could be experimentally targeted, might be required for the conversion between the two modes of reproduction. It is not clear, however, whether the switch between hermaphroditism and gonochorism can occur back and forth since those hermaphroditic species (*P*. sp. 10, *P. entomophagus*, *P. maupasi*), which connect to internal ancestral nodes, could have developed hermaphroditism after divergence from a gonochoristic ancestral species. One might speculate that hermaphroditic lineages are more likely to go extinct than gonochoristic ones. A similar finding was made in the genus *Caenorhabditis*, where hermaphroditism of *C. elegans *and *C. briggsae *has evolved independently [35]. However, based on the available data, the total number of hermaphroditic *Caenorhabditis *species seems to be smaller than in *Pristionchus*.

### Biogeography

One conspicuous observation emerging from the phylogenetic tree reconstructions is the apparent biogeographic distribution of *Pristionchus *species. Species from different geographic origins tend to form clades, such as the European and North American clades or all Asian species assuming a deeper position (Figure 3). The first and most striking observation is the grouping of all endemic North American species into a single highly supported clade. This monophyletic group includes *P. aerivorus*, *P. pseudaerivorus*, *P. americanus*, *P. marianneae*, *P. pauli*, and *P*. sp. 3. These species, which are morphologically very similar but can be distinguished by molecular analysis, are associated with scarab beetles (mainly *Phyllophaga *spp. and *Popilia japonica*) and occur in the same areas in the Eastern US. In the combined CDS of 27 ribosomal protein genes they exhibit 1.7–5.7% nucleotide difference. Surprisingly, the only hermaphroditic species in this clade, *P. maupasi*, a sibling species to *P. aerivorus *and *P. pseudaerivorus*, is widespread in Europe but absent in North America [2,3]. These results suggest that *P. maupasi *has American ancestry and translocation to the European continent and conversion to hermaphroditism may have been associated with its speciation.

North America is the home of the two most closely related species, which differed in their ribosomal protein genes in less than 6% of their coding nucleotides. The two sister species, *P. aerivorus and P. pseudaerivorus*, have even been shown to mate under laboratory conditions to produce sterile offspring that follows Haldane's rule [3,36]. The close relatedness of the members of the North American species group raises the possibility that occasionally they still may hybridize and yield fertile offspring leading to introgression events. This is demonstrated by incomplete and stochastic lineage sorting of the segregating ribosomal protein alleles and by variable molecular markers that are shared between species (data not shown). Thus, certain *Pristionchus *nematodes could prove to be a powerful system to study recent speciation events.

The species, *P. lheritieri*, *P. entomophagus*, *P. uniformis*, *P*. sp. 4, *P*. sp. 6, and *P*. sp. 13 cluster together and form a European branch within the phylogram. Single isolates of *P. entomophagus *from North America could have originated from sporadic dispersal from Europe by beetles or by humans. *P. uniformis *is associated with the CPB and represents the only available *Pristionchus *species with a non-scarab beetle association. *P. uniformis *occurs in Europe and North America and we have previously argued that *P. uniformis *might have invaded Europe together with its insect vector in the late 19^th ^century [3]. However, the phylogenetic position of *P. uniformis *in the middle of the European clade questions this original assumption. Also, *P*. sp. 13 is the sister species to *P. uniformis *within the European clade, providing additional evidence for a European origin of the CPB-associated *P. uniformis*. We are currently collecting *P. uniformis *strains from many additional locations in Europe and North America to study the origin of this species using molecular diversity analyses and relate these studies to similar investigations of the CPB [37]. Finally, it should be noted that *P*. sp. 6, which was found only in the Tübingen area in Germany, lacks a sister species and is the most diverged species in the European clade.

The satellite organism *P. pacificus *seems to be globally dispersed and was sporadically discovered in various soil samples and associated with scarab beetles in the United States, South Africa, China, India, Madagascar, Poland, Montenegro, and Turkey, but predominantly in Japan, where it is closely associated with the Oriental beetle *Exomala orientalis *(Table 1) [2,3,19]. Interestingly, *P. pacificus *is the only *Pristionchus *species currently available with a cosmopolitan distribution. In contrast, its sister species *P*. sp. 11 has been so far isolated only in soil samples from Japan. This clade appears to be basal to the North American and European clades. The other Asian species found in Nepal (*P*. sp. 10) and Japan (*P*. sp. 14, *P*. sp. 15) all are highly diversified from each other and assume basal positions in the phylogenetic tree. Several circumstances could explain these observations. First, samplings in Europe and North America with several hundreds of *Pristionchus *strains were more comprehensive than corresponding samplings in Asia. Therefore, important sister taxa might still be missing in the Asian clade(s), which, if available, might generate a less diversified phylogeny. Second, samplings on the missing continents, Africa, South America and Australia, might provide additional material with important phylogenetic information. Finally, *Pristionchus *nematodes might have originated in Asia and therefore explain the deeper branches. However, such claims await a detailed analysis of beetles and nematodes of the still missing continents, in particular central Africa and South America.

### Biodiversity

In the course of our surveys of the distribution of *Pristionchus *nematodes we could demonstrate that the *SSU *serves as a fast and reliable barcoding marker for species identification with sufficient resolution within the genus. The importance of a fast identification procedure as shown above, which distinguishes morphologically very similar species, is emphasized by the observation of the frequencies of the different *Pristionchus *nematodes in the isolates. Whereas in the total sample size of more than 15,000 beetles and soil samples some species were easily detectable since they occurred several hundred times or were present in at least moderate frequencies, seven species were found by serendipity only in single or very few isolates (Table 1). *Pristionchus *sp. 3 is represented by a single strain isolated from a soil sample from Cold Spring Harbor (New York), *Pristionchus *sp. 11 was found twice in soil samples from Japan, *Pristionchus *sp. 14 was isolated once and *P*. sp. 15 twice from Oriental beetles in Japan. Two isolates of *P*. sp. 10 were obtained from soil samples from Nepal, two isolates of *P*. sp. 4 were found on beetles in western Europe, and *P. americanus *was detected three times on North American beetles [2,3]. Thus, 98% of all analyzed *Pristionchus *isolates belong to only 11 species, the remaining seven species were found only sporadically (Table 1). Rare species were usually found at sampling sites that were also abundant for the more common species showing a total of up to six species in a sampling area. Such patterns were observed on all continents and in all phylogenetic clades. This observation has important consequences for biodiversity assessments. First, it indicates that species asymmetry is a widespread phenomenon in *Pristionchus *and maybe other nematodes as well. Second, the biodiversity of taxa that are as poor in morphological characters as *Pristionchus *nematodes can best be assessed by using molecular tools. Therefore, a fast, reliable and easy barcoding procedure is important for both, species determination and biodiversity and as such complements taxonomy, phylogenetics and population genetics [38].

Besides these immediate implications, future research will address the potential co-evolution between *Pristionchus *nematodes and scarab beetles. Coevolutionary processes are subject to detailed investigations and require knowledge of the phylogeny of both groups of organisms taking part in the coevolutionary process [39]. While the molecular phylogeny of the Coleoptera and scarab beetles is well defined at the higher taxonomic level, lower level phylogeny, necessary for the investigation of coevolutionary processes, is not yet available [40]. Given, the higher number of scarab beetle taxa in all biogeographic groups, in particular Asia and North America, such an analysis will be time intensive.

## Conclusion

We present an approach based on concatenated ribosomal protein genes to reconstruct a robust phylogenetic framework of the genus *Pristionchus*, which represents the basis for evolutionary interpretations of developmental, behavioral and ecological patterns. The phylogenic tree indicates distinct but closely related species, which group into clades that correspond largely with their geographic origin. Hermaphroditism has evolved independently in five *Pristionchus *species suggesting a frequent conversions toward hermaphroditism. Our studies also indicate the use of *Pristionchus *for nematode biodiversity assessments. Ninety-eight per cent of the 1,200 analyzed *Pristionchus *isolates represent the 11 most common species, whereas the other seven species are represented by small numbers of isolates. Such patterns indicate that species asymmetry is a widespread phenomenon that can best be investigated by molecular tools.

## Methods

### Isolate and strain definitions

We use the following definitions to distinguish "isolates" and "strains". An isolate is an isogenic female line, which is derived from a beetle sample and subjected to molecular and experimental analysis. After species identification we established one isolate per species and location as a strain. The strains are permanently cultured in the lab, have a strain number and are also kept as frozen stocks. For each new species designated by molecular sequence analysis and mating experiments, one strain was defined as a reference strain (see below). The phylogenetic analysis described here was carried out by using the reference strains of all available *Pristionchus *species (Table 1).

### Molecular species identification

Species were identified employing the small subunit rRNA gene (*SSU*) [2-4]. In short, genomic DNA from single nematodes was prepared using the NaOH digestion procedure described by Floyd et al. [20]. A single worm was transferred to 20 μl of 0.25 M NaOH, incubated overnight at 25°C and heated to 99°C for 3 min before 4 μl of 1 M HCl, 10 μl of 0.5 M Tris-HCl (pH 8.0) and 5 μl of 2% Triton X-100 were added. The mixture was heated to 99°C for 3 min, frozen at -20°C and reheated at 99°C for further 3 min. Two microliters of this extract were used for subsequent polymerase chain reaction (PCR).

A 1 kb fragment of the *SSU *was amplified by PCR using the primers SSU18A (5'-AAAGATTAAGCCATGCATG-3') and SSU26R (5'-CATTCTTGGCAAATGCTTTCG-3') [8,20]. The reactions were performed in 25 μl of 1× PCR buffer (Amersham Biosciences, Freiburg, Germany) containing 2.5 mM of MgCl_2_, 0.16 mM of each deoxynucleoside triphosphate, 0.5 μM of each primer, 2 μl of the lysate, and 2 units of *Taq *DNA polymerase (Amersham Biosciences). The reactions were started by initial denaturation at 95°C for 2 min in a T gradient thermocycler (Biometra, Göttingen, Germany), followed by 35 cycles of denaturation at 95°C for 15 sec, primer annealing at 50°C for 15 sec, and extension at 72°C for 2 min. A final incubation step at 72°C for 7 min concluded the reaction. For sequencing of approximately 500 bp of the 5'-terminal end of the *SSU *using the primer SSU9R (5'-AGCTGGAATTACCGCGGCTG-3') one microliter of a 10 to 20 fold dilution of the PCR mixes was directly added to the Big Dye terminator sequencing mix (Applied Biosystems, Darmstadt, Germany). A BLAST search option of *Pristionchus SSU *sequences will be set up on our web site [18,41].

### Mating experiments

To support the species molecular identification of a novel isolate we performed mating experiments with the reference strain of the respective species (see below for definitions). Five virgin females were put on a plate with a small spot of *E. coli *OP50 together with five males of the reference strain of a certain species. On a second plate we picked the opposite sexes of the two strains to test for reciprocity. If there was no offspring after one week, the experiments were repeated two more times. If fertile offspring occurred we considered the two strains to belong to the same species.

### Construction and sequencing of expressed sequence tag (EST) libraries

To search for abundantly expressed genes with similar expression patterns in different nematode species, total RNA from 50 to 100 μg of ten nematode reference strains was isolated using the TRIZOL reagent (Invitrogen) following the manufacturer's instructions. The reference strains were PS312 (*Pristionchus pacificus*), SB245 (*P. lheritieri*), RS0141 (*P. uniformis*), RS0143 (*P. maupasi*), RS0144 (*P. entomophagus*), RS5050 (*P*. sp. 4), RS5101 (*P*. sp. 6), RS5106 (*P. aerivorus*), CZ3975 (*P*. sp. 3) and RS1982 (*Koerneria *sp.) as outgroup genus closely related to *Pristionchus*. Two microliters of total RNA were used for double-stranded cDNA synthesis with the help of the BD SMART PCR cDNA synthesis kit (Becton Dickinson, Heidelberg, Germany) and the BD Advantage 2 Polymerase mix (Becton Dickinson) according to the manufacturer's instructions with the following exceptions. The 3' primer used for first strand cDNA synthesis was RH5620 (5'-GAAGATCTAGAGCGGCCGCCCTTTTTTTTTTTTTTT-3'), the 5' primer was RH6478 (5'-GGTTTAATTACCCAAGTTTGAGCGGG-3'). The sequence of the 5' primer is derived from the conserved SL1 leader of *Pristionchus *and was chosen to specifically amplify the first gene of trans-spliced polycistronic mRNA. Second-strand synthesis was performed by 17 PCR cycles with the primers RH6479 (5'-AGTGTCGACGGTTTAATTACCCAAGTTTGAG-3') and RH6295 (5'-GAAGATCTAGAGCGGCCGCCC-3') using an annealing temperature of 64°C. Double-stranded cDNA fragments were purified using QIAquick columns (Qiagen, Hilden, Germany). Single deoxyadenosyl overhangs were added by incubating the DNA in *Taq *reaction buffer with *Taq *DNA polymerase (Amersham Biosciences) in the presence of 0.2 mM ATP at 37°C for 15 min before ligation into the pCR2.1-TOPO vector (Invitrogen, Karlsruhe, Germany). The DNA was electroporated in OneShot *E. coli *cells (Invitrogen) and plated onto LB plates containing 50 μg of ampicillin. For insert sequencing 96 to 384 single colonies were picked, plasmid DNA was extracted using the QIAprep 96 Turbo BioRobot Kit (Qiagen). Sequencing reactions were performed with the BDT V 3.1 reaction mix using the SL1-specific primer BJ234 (5'-GGTTTAATTACCCAAGTTTGAG-3').

### EST analysis and gene-specific primer design

The proteins encoded by the SL1-transspliced genes in the initial EST screen were identified by BLASTX searches of WormBase and the non-redundant database at GenBank [42-44]. Sequences of selected genes were aligned manually with the help of the Seqpup 0.6 f software for Macintosh. Sequence stretches conserved between *Pristionchus *and *Koerneria *sp. were chosen to design gene-specific generic RT-PCR primers (Additional file 1). The primers were checked for secondary structures and compatibility to the SL1-primer BJ234 and the oligo(dT)-primer RH5620 with the help of the OLIGO 4.0 software (MedProbe, Oslo, Norway).

### RT-PCR of ribosomal proteins genes

RNA was reverse transcribed into cDNA with the help of the Omniscript reverse transcriptase kit (Qiagen, Hilden, Germany) and the primer RH5620 (s. construction of EST libraries). Complete transcripts were synthesized by RT-PCR in two overlapping fragments per gene. The SL1-specific primer BJ234 (5'-GGTTTAATTACCCAAGTTTGAG-3') was used together with the gene-specific antisense primers to obtain the 5' parts of the transcripts and the combination of RH5620 and the sense primers to obtain the 3' parts. The PCR was performed with the help of the HotStar *Taq *Plus DNA polymerase kit (Qiagen) including 1× Q solution in the reaction mix. Conditions were initial activation of the enzyme at 95°C for 5 min, followed by 40 cycles of denaturation at 94°C for 30 sec, primer annealing at 50°C for 30 sec, and primer extension at 72°C for 3 min. The reaction was completed by incubation at 72°C for 10 min. PCR fragments were electrophoresed through an 1.5% agarose gel, purified using the Wizard SV gel purification kit (Promega) and sequenced directly using the respective PCR primers. The sequences have been deposited to the GenBank database and can be retrieved under the Accession Numbers [GenBank:EF634493-EF635005].

### Phylogenetic analyses of ribosomal protein genes

Sequences were aligned manually using the Seqpup 0.6 f software for Macintosh [45]. The alignments were inspected for the presence of coding open reading frames, which were then confirmed by comparison to the *C. elegans *and *C. briggsae *orthologues. Genes that were either duplicated in *Caenorhabditis *or appeared to be present in more than one copy in *Pristionchus *were omitted from the analysis. Reliable coding sequences (from codons 3 to 8, depending on the gene, through the termination codon) were concatenated and subjected to analysis by Modeltest 3.7 to determine the best-fit nucleotide substitution model for maximum likelihood analysis (ML) by likelihood ratio tests (LRT) using the Akaike information criterion [27,46]. Only *Pristionchus *sequences were used for parameter determination. The selected settings corresponded to the GTR+G+I model with 6 substitution types (A-C = 0.6105, A-G = 1.3696, A-T = 1.1141, C-G = 0.9545, C-T = 6.1059, G-T = 1.0000), assumed nucleotide frequencies of A = 0.25540, C = 0.31530, G = 0.25700, T = 0.17230, assumed proportion of invariable sites I = 0.5307, rates at variable sites following the γ distribution with shape parameter α = 0.7072.

The phylogenetic reconstruction was performed by the heuristic search algorithm of the PAUP*4.0b10 software using the parameters of the selected substitution model and the likelihood optimality criterion [28]. The branch-swapping algorithm was set to tree-bisection-reconnection (TBR). Neighbor joining (NJ) trees using the BIONJ method and maximum parsimony (MP) trees were drawn by the same program [29,47]. Alignment gaps were eliminated from the analysis. All resulting trees showed the same topology. Support for the tree topology was obtained by 1000 bootstrap replications using the NJ or MP algorithms [48]. Likelihood mapping of individual nodes was performed with the help of the program TREE-PUZZLE 5.2 [32,33]. Additional support for the tree topology was obtained by Bayesian analysis using the MrBayes 3.2.1 software for Macintosh [31]. The evolutionary model was set to GTR+I+G. The analysis was run for 150,000 generations with a burn-in time of 37,500 generations and a sampling frequency of 1 in 100 generations. Frequencies of synonymous and non-synonymous substitutions were determined using the MEGA 3.1 software [24].

## Abbreviations

AIC Akaike information criterion

CDS Coding sequence

CPB Colorado potato beetle

EST Expressed sequence tag

ML Maximum likelihood

MP Maximum parsimony

NJ Neighbor-joining

*SSU *small subunit ribosomal RNA gene

## Authors' contributions

WEM generated isogenic female lines, designed and carried out all of the molecular experiments, performed all of the phylogenetic analysis and wrote the manuscript. MH carried out all of the fieldwork, generated isogenic female lines and did the mating experiments. RJS designed and coordinated the experiments and wrote the manuscript. All authors read and approved the final manuscript.

## Supplementary Material

Additional file 1 Oligonucleotides used in this study. Oligonucleotides used for cDNA synthesis, RT-PCR and sequencing are shown. a, sense; as, antisense; cDNA, cDNA synthesis; seq, sequencing. Click here for file
