# Supplementary material for: Phylogeny of the nematode genus Pristionchus and implications for biodiversity, biogeography and the evolution of hermaphroditism
Source: BMC Evol Biol. 2007 Jul 2;7:104. doi: 10.1186/1471-2148-7-104 (PMC1929057; doi:10.1186/1471-2148-7-104)
Supplement: Additional file 1 — Oligonucleotides used in this study. Oligonucleotides used for cDNA synthesis, RT-PCR and sequencing are shown. a, sense; as, antisense; cDNA, cDNA synthesis; seq, sequencing. [file 1471-2148-7-104-S1.doc]

Oligonucleotides used in this study.

_______________________________________________________________________________________

Designation Gene Strand Purpose Sequence (5’->3’)

_______________________________________________________________________________________

BJ234 *SL1* s cDNA/seq GGTTTAATTACCCAAGTTTGAG

SSU18A *SSU* s PCR AAAGATTAAGCCATGCATG

SSU26R *SSU* as PCR CATTCTTGGCAAATGCTTTCG

SSU9R *SSU* as seq AGCTGGAATTACCGCGGCTG

RH5620 oligo(dT)-tail as SMART cDNA GAAGATCTAGAGCGGCCGCCC(T)_15_

RH6478 *SL1*-derived s SMART cDNA GGTTTAATTACCCAAGTTTGAGCGGG

RH6479 *SL1*-derived s SMART cDNA AGTGTCGACGGTTTAATTACCCAAGTTTGAG

RH6295 modified RH5620 as SMART cDNA GAAGATCTAGAGCGGCCGCCC

WM8115 *rpl-1* s PCR/seq CCTGCATGAACGCYGAYGACCTC

WM8116 *rpl-1* as PCR/seq CTGGAACTTGATGGTVGCCTTGA

WM11157 *rpl-1* s PCR/seq TCGGTCCTGGWCTCAACAAG

WM12125 *rpl-1* as PCR/seq GAGAGGGAGATGTTGGAGACGAG

WM12738 *rpl-1* as PCR/seq TTCCGACGGAGAGGCAGAGCAC

WM10292 *rpl-2* s PCR/seq CGAGTTCCGYTGCCCBTACAAG

WM10293 *rpl-2* as PCR/seq CAATRTGCTGGTGGTTACCTCC

WM12261 *rpl-2* s PCR/seq CACAACCCCGACACCAAGAAGA

WM8117 *rpl-10* s PCR/seq CTCGTCAAGAACTGCGGAAAGGA

WM8118 *rpl-10* as PCR/seq AGRCGRACGGAGAAGAGGAARTC

WM8568 *rpl-10* as PCR/seq GTAGGCACCACGCATTCCAGTC

WM9621 *rpl-10* s PCR/seq CCAACAAGTTCCTCGTCAAG

WM9622 *rpl-10* as PCR/seq TCGGCTCTCATTCTCTCGTA

WM8066 *rpl-14* as PCR/seq CGRTCGAAGTCSGWGAGRGTVGA

WM8067 *rpl-14* s PCR/seq CAAGTTCAAGGTCCCMATYAGA

WM8898 *rpl-14* as seq GCAGCDAGCTTCTTDGCCCA

WM11060 *rpl-14* as PCR/seq AATCTGTTTCTGGCYTGCTT

WM12126 *rpl-14* s PCR/seq TGGTAGAAATCGGYCGTGTSGT

WM12127 *rpl-14* as PCR/seq AGTCTRATRGGGACCTTGAACT

WM10290 *rpl-16* s PCR/seq CAACCCSAGACGCGGACCTTTC

WM10291 *rpl-16* as PCR/seq GGCTCCCTTCACCTTTCTCTTG

WM12128 *rpl-16* as PCR/seq TGATGGCGGAGGGGAGGACAAG

WM10288 *rpl-23* as PCR/seq GTCCCTTCATTTCACCCTTGTT

WM10289 *rpl-23* s PCR/seq TWCGTGGAAGACTYAACAGACT

WM12129 *rpl-23* s PCR/seq GCCGGAGCTCAGGAAGAAGGTC

WM12169 *rpl-23* s PCR/seq GCGTCCGTCAAGAAGGGCAAGC

WM8220 *rpl-26* s PCR/seq TCGACAACGACAGAAAGAAGA

WM8221 *rpl-26* as PCR/seq ACGGAGTCRTCGCTGTRCTTGC

WM10879 *rpl-26* s PCR/seq GATGTCYGCTCCCCTCACCA

WM12708 *rpl-26* as PCR/seq CATCTACTCCTGCACGTCATCG

WM8203 *rpl-27* s PCR/seq ATGGGAAAGAMGACGCAGACT

WM8204 *rpl-27* as PCR/seq GYGCTCTTCTCTTCTTCTTGG

WM8896 *rpl-27* as seq CTTRABRAARGGCTTCAGCT

WM8897 *rpl-27* s seq GAGAGGAACAAGCTGAAGCC

WM12167 *rpl-27* s PCR/seq GTCGTTCTCGTCCTCAGAGGAA

WM12168 *rpl-27* as PCR/seq TTCTCTTCTTCTTGGGCTCCTT

WM12260 *rpl-27* as PCR/seq TGCCGGCCTTGTGCTTCTCCTC

WM8205 *rpl-27a* s PCR/seq GGTCAYGGTCGTGTGGGTAAGCA

WM8206 *rpl-27a* as PCR/seq AGTCCCTTHCCGAGVACCTTGAA

WM8263 *rpl-28* s PCR/seq CCGTCAGCGYGGMATCCAGAAG

WM8264 *rpl-28* as PCR/seq GCTGGASGGAGCGGAGRAGCTG

WM10880 *rpl-28* as PCR/seq ACGWCGWGCGGCGAGCTT

WM12706 *rpl-28* s PCR/seq GTCAGCGTGGAATCAACAAGCA

WM12707 *rpl-28* s PCR/seq TAACCTCAAGAGCATCAACAGC

WM7991 *rpl-29* s PCR/seq CASYCAYAACCAGAACCGCAAGGA

WM7992 *rpl-29* as PCR/seq TCTTGTYGTGCTTCTTGGCGAA

WM8111 *rpl-30* s PCR/seq AGCAGAAGAARTCGGCGGAGAA

WM8112 *rpl-30* as PCR/seq GCTCGATGTTGTTDCCRTTGTA

WM12705 *rpl-30* s PCR/seq TCGGATACAAGCAGACCCTCAA

WM10286 *rpl-31* s PCR/seq TWCACGATYAACCTCCACAAGA

WM10287 *rpl-31* as PCR/seq TARAGCTTGYGRGGTGAGTCCT

WM10881 *rpl-31* as PCR/seq GAGCTTGYGRGGTGAGTCCT

WM8207 *rpl-32* s PCR/seq AACCGYGTCCGTCGTCGYTTCAA

WM8208 *rpl-32* as PCR/seq GVGACGGCGTGBGCGACCTCTC

WM8817 *rpl-32* as PCR/seq TCTTGTTGTTCATGAGGAGCAT

WM8895 *rpl-32* as seq GGGCGTGGCCGTTGGTCAGTCG

WM12704 *rpl-32* s PCR/seq ACTACCGTCTGAAGCCCAACTG

WM10284 *rpl-34* as PCR/seq TTTATTTGTCGGCGTTCTGCTT

WM10285 *rpl-34* s PCR/seq TACAACACYGCSTCCAACAAGA

WM10282 *rpl-35* s PCR/seq GATYAGAGTSGTCCGCAAGAAC

WM10283 *rpl-35* as PCR/seq AGGTACTKCTTKCCCTTGTAGA

WM8218 *rpl-38* s PCR/seq CCCGCAGAAAGGACGCCAAGT

WM8219 *rpl-38* as PCR/seq TSTCGGCCTTYTCCTTRTCCT

WM8265 *rpl-39* s PCR/seq GCCAAGGCCCAGAAGCAGAACC

WM8266 *rpl-39* as PCR/seq AGCTTCAGCTTGGTRCGTCTCC

WM10280 *rps-1* s PCR/seq TAAGAAGGGAGCCARGAAGAAG

WM10281 *rps-1* as PCR/seq AATCTTGACYTTVCGGATGTAC

WM10884 *rps-1* s seq CGCAAGTTCCGTCTCATCTG

WM10885 *rps-1* as seq ACCATGGTGTGCCACTTCTT

WM12262 *rps-1* as PCR/seq AAGACATGCCGTGGAAGTTGGT

WM12263 *rps-1* s PCR/seq CCTCCAAGAGACAGCACCAAGT

WM8113 *rps-14* as PCR/seq GGRGTCTTNGTTCTRGTTCCTC

WM8114 *rps-14* s PCR/seq GCYCAYATYTTCGCYTCTTTCAA

WM8569 *rps-14* as PCR/seq TTGATGTGCAGAGCGTTGATAC

WM10274 *rps-20* as PCR/seq ACCTGGTGGAGCACGTCCGA

WM10275 *rps-20* s PCR/seq CGCATCACCACCCGCAAGAC

WM10272 *rps-21* s PCR/seq CTCTACATYCCCCGCAAGTG

WM10273 *rps-21* as PCR/seq GCAGATGGCGTAGCGGTTGA

WM8267 *rps-24* s PCR/seq ASAAGCTCGCCTCTCTGTACAA

WM8268 *rps-24* as PCR/seq GCCTTCTTCTGTCTGTTCTTT

WM8894 *rps-24* s seq CTAGACCACCCCCGACCTTGTT

WM8269 *rps-25* s PCR/seq GARARGCMAAGAAGAAGAAGTG

WM8270 *rps-25* as PCR/seq CRGAGGGWGTGATVAGCTTGTA

WM11295 *rps-25* s PCR/seq GGTAAGAAYCCYCCYAAGAAG

WM7993 *rps-27* s PCR/seq AYCCYAAYTCSTTCTTCATGGA

WM7994 *rps-27* as PCR/seq GGYTTARTGCTGCTTCTTYCTGAA

WM7995 *rps-27* as PCR/seq AGTTTAGTGGGCCTTCTTTCTGAA

WM12703 *rps-27* as PCR/seq CATCCGACGCAGACGACGACAG

WM8815 *rps-28* s PCR/seq TCGCTAAGGYCACCAAGATCCT

WM8816 *rps-28* as PCR/seq GGCCCTTRACGTTGCGGATGAT

WM11061 *rps-28* s PCR/seq CGCACTGGATCYCAGGGACA

WM10278 *rps-8* s PCR/seq TCTACAAYGCSTCCAACAAC

WM10279 *rps-8* as PCR/seq TCCTCCTCCTCSGTGAYCTT

WM10882 *rps-8* s seq GTGCCYTTCCGTCAGTGGTA

WM10975 *rps-8* as seq GNCTCRTACCACTGACGGAA

WM12286 *rps-8* as PCR/seq TGTTCTTCACRAGGGTCTTGGT

*_________*_____________________________________________________________________________

a, sense; as, antisense; cDNA, cDNA synthesis for EST libraries; seq, sequencing
